# Sperm function *in vitro* and fertility after antibiotic-free, hypothermic storage of liquid preserved boar semen

**DOI:** 10.1038/s41598-019-51319-1

**Published:** 2019-10-14

**Authors:** Dagmar Waberski, Anne-Marie Luther, Benita Grünther, Helen Jäkel, Heiko Henning, Charlotte Vogel, Wolfgang Peralta, Karl Fritz Weitze

**Affiliations:** 1 Unit for Reproductive Medicine of Clinics/Clinic for Pigs and Small Ruminants, University of Veterinary Medicine Hannover, Bünteweg 15, D-30559 Hannover, Germany; 2 Department of Equine Sciences, Faculty of Veterinary Medicine, Utrecht University, Yalelaan 112, 3584 CM Utrecht, The Netherlands; 3 Institute for Biometry, Epidemiology and Information Processing (IBEI), University of Veterinary Medicine Hannover, Bünteweg 2, D-30559 Hannover, Germany; 4 Agricola Super Ltda., Rancagua, Chile

**Keywords:** Animal biotechnology, Cell death

## Abstract

The role of antibiotics (AB) in semen extenders as a potential contribution to the global antimicrobial resistance threat is emerging. Here, we establish an AB-free hypothermic preservation strategy for boar semen and investigate its impact on sperm function, microbial load and fertility after artificial insemination (AI). Spermatozoa (12 boars) preserved in AB-free AndroStar Premium extender at 5 °C maintained high motility, membrane integrity, and a low DNA-fragmentation index throughout 72 h storage and results did not significantly differ from controls stored at 17 °C in extender containing AB (p = 0.072). Likewise, kinetic response of spermatoza to the capacitation stimulus bicarbonate during 180 min incubation in Tyrode’s medium did not differ from 17 °C-controls. In a competitive sperm oviduct binding assay, binding indices did not differ between semen stored for 72 h AB-free at 5 °C and 17 °C-controls (n = 6 boars). Bacterial load < 10^3^ CFU/ml after 72 h was measured in 88.9% of samples stored at 5 °C AB-free compared to 97.2% in 17 °C-controls (n = 36 semen pools, 23 boars). Fertility traits of 817 females did not differ significantly between the two semen groups (p > 0.05). In conclusion, a hypothermic semen preservation strategy is presented which offers antibiotic-free storage of boar semen doses.

## Introduction

Artificial insemination (AI) in pigs is a highly efficient assisted reproductive technology used in more than 90% of sows in the primary pork producing countries. The main benefit of AI in domestic animal species is seen in the prevention of transmission of venereal diseases and an accelerated genetic progress^1^. Semen from healthy, fertile boars and similarly from other species contains bacteria stemming from natural colonization in the male tract and from the environment. The majority of contaminants are Gram-negative bacteria, belonging to the *Enterobacteriaceae* family^2^. Storage of boar spermatozoa at a relatively high temperature (16 to 18 °C) in a nutrient-rich preservation medium favors bacteria growth in semen doses. Storage at lower temperature, however, is contraindicated by the pronounced cold shock sensitivity of boar spermatozoa which has been associated with a high content of polyunsaturated fatty acids and a low sterol to phospholipid ratio of sperm membranes^3^. Accordingly, frozen semen is not routinely used in commercial farms for market pig production. To prevent disease transmission in sows through insemination and harmful effects of bacteriospermia on sperm quality^4,5^, the addition of antibiotics to semen extenders is mandatory in the European Union and commonly used in other countries^6^. As antimicrobial resistance poses a serious global threat of growing concern to human health and economics^7,8^, awareness increases that antimicrobials added to semen extenders may represent a route for dissemination of antimicrobials and antimicrobial resistant bacteria into the environment^9^. In particular, the natural back-flow of 70 to 90% of the semen dose volume (30–80 ml) from the sow’s genital tract for a period of two hours after insemination^10,11^ leads to a consistent entry of antibiotics and bacteria into liquid manure causing a subsequent risk of entry in the human food chain^12,13^. Moreover, the permanent use of various antibiotics and disinfectants in AI centers favors the generation of multidrug-resistant bacteria^2,14^, often belonging to the main nosocomial pathogens^15^.

As part of the global antimicrobial resistance defense strategy, alternative strategies for bacterial control in extended boar semen are being studied. Antibiotic-free storage of semen doses would be a primary choice. Physical reduction of bacterial contamination before semen storage can be achieved by low density colloid centrifugation^16^ or microfiltration^17^ but is still limited for practicable use due to the low laboratory efficiency. Bacteriostasis by hypothermic semen storage could be another option if chilling-induced thermotropic phase transitions of membrane lipids leading to structural and metabolic disruption of sperm function^18^ could be minimized. In an earlier study, a threshold for storage temperature of 12 °C was reported before motility and fertility were affected^19^. Accordingly, earlier attempts to preserve boar semen at 4 or 5 °C resulted in decreased motility, membrane integrity, mitochondrial activity and chromatin stability^20–23^. Preliminary studies using commercial membrane protecting semen extenders AndroStar Plus^24^ or Androstar Premium extender^25^ (Minitüb, Tiefenbach, Germany) indicate that low-temperature storage in absence of antibiotics could be an option for boar semen preservation if maintenance of sperm quality and control of bacterial growth is achieved. Recent research demonstrated that cell alterations in preserved boar semen can be diminished by isothermic one–step dilution at 30–33 °C followed by slow cooling^26^, a minimum sperm concentration of 15 × 10^6^/ml^27^, use of modern commercial semen extenders^28^ and motionless semen storage^29^.

With consideration of the above, we hypothesize that implementation of all semen processing steps at their currently known optimal level offers the opportunity to antibiotic-free hypothermic storage of boar semen without compromising fertility. A holistic approach is used which includes the screening of bacteria counts in semen extender media, the use of sensitive *in vitro* and *ex vivo* measures to assess the sublethal alterations in sperm function, and ultimately a highly standardized *in vivo* fertilization test under field conditions. Overall, the goal of the current study is to investigate the possibility of using low temperature storage of boar semen in absence of antibiotics.

## Material and Methods

### Reagents and media

Chemicals were of analytical grade and, unless otherwise stated, purchased from Sigma-Aldrich (Steinheim, Germany). Fluorochromes were obtained from Life Technologies (Darmstadt, Germany), and semen extender media were obtained from Minitüb (Tiefenbach, Germany).

### Temperature dependence of bacterial growth in semen extender

In a pre-experimental screening, the influence of temperature on bacterial growth during culture at 5 °C and 17 °C was examined. Bacterial counts (CFU/ml) were evaluated during a storage period for 72 h in either BTS semen extender consisting of 205 mM glucose, 20.4 mM Na_3_C_6_H_5_O_7_, 10.0 mM KCL, 15 mM NaHCO_3_, 3.36 mM EDTA or culture medium consisting of 85.5 mM NaCl, 39 mM NaOH in 1000 µl aqua dest. supplemented with 10 g peptone and 10 g meat extract. Typical mesophilic bacteria isolated from boar semen, *Pseudomonas aeruginosa*, *Serratia marcescens, Klebsiella oxytoca* and *Burkholderia species* were evaluated. Sterile-filtered extender and culture media were inoculated with 3 × 10^3^ CFU/ml of single bacteria strains. After aerobic culture in blood agar for 48 h at 37 °C, colony forming units (CFU) per ml were counted.

### *In vitro* trials: Sperm quality in stored semen samples

#### Animals, semen processing and storage

All procedures that involved animals were carried out in accordance with guidelines and regulations according to the European Commission Directive for Pig Welfare and were approved by the institutional animal welfare committee of the University of Veterinary Medicine Hannover. Entire ejaculates were collected by the gloved-hand method from twelve fertile, sexually mature boars (1.5 to 6 yr of age) of different breeds (Piétrain, German Landrace, Large White, Duroc). Normospermic ejaculates were extended isothermically (37 °C) on split-sample basis in order to prepare four split semen sample groups according to extender type and storage temperature using: (1) the short-term extender Beltsville Thawing Solution^30^ with antibiotics (AB; gentamicin sulphate (0.25 g/L), storage at 17 °C (17 °C, BTS); (2) BTS extender with AB, storage at 5 °C (5 °C, BTS); (3) the long-term extender Androstar Premium (APrem) with antibiotics (gentamicin sulphate (0.25 g/L), storage at 17 C° (17 °C, APrem); or (4) APrem without antibiotics, storage at 5 C° (5 °C, APrem). Aliquots of samples diluted in BTS were used as positive control for 17 °C semen storage and as negative control for 5 °C storage. Final sperm concentration in plastic semen tubes (90 ml) was 20 × 10^6^ sperm /ml. A subset of samples extended in BTS and samples of APrem with AB were maintained at 22 °C for 90 min and then stored at 17 °C in the dark. A further subset of samples extended in BTS and samples extended APrem without AB were placed in a cardboard box with a lid together with isothermic (28 °C) water–filled tubes. Boxes with 35 tubes were subsequently kept for 6 h at 22 °C and then stored in a refrigerator at 5 °C in the dark.

#### Computer-assisted semen analysis

Sperm kinematics was objectively evaluated after 24 h, 72 h, and 144 h with the CASA system AndroVision, Version 1.1 (Minitüb, Tiefenbach, Germany), using pre-warmed four-chamber slides (Leja, Nieuw Vennep, NL) with a chamber depth of 20 µm. For each sample, six successive fields in the central axis of a chamber were recorded at a rate of 30 frames per 0.5 s for each field using a 100 × magnification. At least 500 spermatozoa per sample were analyzed. The sperm kinematic variables recorded were the overall percentage of motile spermatozoa, curve line velocity (VCL µm/sec), beat cross frequency (BCF, Hz) and amplitude of lateral head displacement (ALH, µm). Spermatozoa were evaluated as “motile” by the AndroVision software algorithm when their ALH was higher than 1.0 µm and their VCL was higher than 24.0 µm/sec.

#### Flow cytometry

Sperm membrane integrity was analyzed at 24 h, 72 h, and 144 h with a Galaxy Flow cytometer (DAKO, Hamburg, Germany). A 5 µl subsample of diluted semen was transferred to 995 µl of HEPES-buffered saline medium (HBS: 137 mM NaCl, 20 mM HEPES, 10 mM Glucose, 2.5 mM KOH, 3 mg/ml BSA (Cohn’s fraction V), pH 7.4 at 20 °C, 300 ± 5 mOsmol/kg) including propidium iodide (PI; final concentration 5 µg/mL), fluorescein-isothiocyanate-conjugated peanut agglutinin (PNA-FITC; final concentration 3.0 µg/mL), and Hoechst 33342 (H-342, final concentration 0.75 µg/mL). After incubation at 25 °C in the dark for 15 min, 10,000 events were analyzed by ‘FloMax’ software (v2.4, Partec, Münster, Germany). Fluorochromes were excited at 488 nm (argon ion laser, 20 mW) and at approx. 365 nm (mercury lamp). Signals were detected using a 537.5/22.5 nm filter (PNA-FITC), a 630 nm LP filter (PI), and a 455/10 nm filter (H-342). Membrane intact spermatozoa were those with intact plasma membranes and acrosome (Hoechst-positive, PI negative and PNA-FITC negative).

The DNA fragmentation index (DFI) of spermatozoa was assessed using the sperm chromatin structure assay (SCSA^31^) in semen samples stored for 72 h. The assay tests for the resistance of spermatozoa to acid denaturation *in situ*. Snap-frozen extended semen aliquots were thawed and 10 μl were added to 490 μl TNE buffer (0.15 M NaCl, 0.01 M TRIS-HCL, 1 mM disodium EDTA, pH 1.2). Then, an aliquot of 200 μl was taken and 400 μL acid solution (0.08 M HCL, 0.15 M NaCl, 0.1% Triton X-100, pH 1.2) was added and samples were vortexed for 30 s in the dark. To stop the denaturation reaction, 1.2 mL acridine orange (Polysciences, Warrington, PA, USA) staining solution (0.15 M NaCl, 0.0037 M citric acid, 0.126 M Na_2_HPO_2_, 0.0011 M disodium EDTA, pH 6.0; containing 6 μg mL-1 acridine orange) was added. Samples were placed on ice for 3 min and then 10,000 cells were analyzed with an average flow rate of 200–300/sec using a FACScan flow cytometer (Becton-Dickinson, Heidelberg, Germany) with a 488-nm laser for excitation, and 530/30-nm band pass and 650-nm long pass filters for detecting green and red fluorescence, respectively. The DNA fragmentation index (DFI) was determined as described by Evenson *et al*.^32^.

The response to capacitating conditions was evaluated by measurement of intracellular calcium as described by Henning *et al*.^33^ in semen samples (n = 6) stored for 72 h. 4 mL of extended semen were incubated with 4 µL of Fluo-3/AM stock solution (final concentration: 1 µM) for 30 min at RT in the dark. Aliquots of Fluo-3-loaded spermatozoa were incubated at a concentration of 1.5–2.0 × 10^5^/mL in two variations of Tyrode´s medium. The capacitating medium Tyrode A consisted of 96 mM NaCl, 20 mM HEPES, 5 mM glucose, 3.1 mM KCl, 2 mM CaCl_2_, 0.4 mM MgSO_4_, 0.3 mM KH_2_PO_4_, 100 µg/mL gentamycin sulphate (SERVA, Heidelberg, Germany), 20 µg/mL phenol red, 1.0 mM sodium pyruvate, 21.7 mM sodium lactate, 3 mg/mL bovine serum albumin (Cohn’s fraction V, fatty acid free), 15 mM NaHCO_3_ and 2 mM CaCl_2_. Additionally, non-capacitating control medium Tyrode C was used to study the specific effect of the capacitating conditions. The control medium was lacking the capacitation inducer bicarbonate. Both media were adjusted to a pH of 7.40 at 38 °C and an osmolality of 300 mOsmol/kg. Propidium iodide (final concentration: 2.5 µg/mL) was added before the beginning of incubation in the Tyrode’s media. Samples were assessed after 3, 60, 90, 120 and 180 min of incubation at 38 °C under CO_2_ (Tyrode) or under air (Control). After 150 min, Ca^2+^ ionophore A23187 was added to induce cytosolic calcium influx as a positive control. The destabilization of spermatozoa under capacitating and non-capacitating conditions was evaluated by flow cytometric measurement of the live sperm population with low intracellular calcium (H-342 positive, PI-negative, Fluo-3 negative).

### *Ex vivo* trial: Sperm-oviduct binding

The sperm’s capacity to form the female sperm reservoir was determined using a recently established competitive oviduct binding assay^34^. Single ejaculates from six boars were diluted with APrem to 50 × 10^6^ sperm/ml. Diluted semen was split into two aliquots and sperm in these subsamples were labelled either with MitoTracker Red FM or MitoTracker Green FM (200 nM) for 15 min at 38 °C in the dark. To remove excessive dye, samples were centrifuged through 20% Percoll. Percoll-saline working solutions (300 ± 5 mOsmol/kg, pH 7.40 ± 0.05) were prepared by diluting Percoll (GE Healthcare, Munich, Germany) with a HEPES-buffered saline solution (HBS; 137 mM NaCl, 20 mM HEPES, 10 mM Glucose, 2.5 mM KOH, pH 7.60 ± 0.05 at room temperature, 300 ± 5 mOsmol/kg) as described previously^35^. Samples were centrifuged for 10 min at 300 g followed by 10 min at 750 g. Sperm concentration in the pellet was adjusted to 20 × 10^6^ sperm/mL with isothermic extender supplemented with 10% (v/v) fresh autologous seminal plasma. Semen was then stored at 5 °C and 17 °C for 72 h, respectively. Oviduct explants were collected from slaughtered sows (parity ≥1). Explants were prepared as described previously^34,36^. Each semen sample was tested with four explants from two sows. Semen samples were pre-incubated at 38 °C in a water bath and then co-incubated for 15 min at 38 °C under 5% CO_2_ in a cross-over design with explants. Red-tagged sperm from 5 °C storage were tested against green-tagged sperm stored at 17 °C on the same explant. Next, the opposite combination was tested with another explant from the same sow. The average sperm binding index for samples stored at 5 °C and 17 °C and the average ratio of bound spermatzoa were calculated.

The average number of bound spermatozoa per 1 mm^2^ was defined as binding index (BI) as described previously^34,36^:

$${\rm{BI}}({\rm{E}}1)=({{\rm{N}}}_{1}+{{\rm{N}}}_{2}+{{\rm{N}}}_{3})/({{\rm{A}}}_{1}+{{\rm{A}}}_{2}+{{\rm{A}}}_{3})$$

where E1 is the explant from Sow no. 1, A_1_, A_2_ and A_3_ are the areas of Location no. 1, 2, and 3, respectively, and N_1_, N_2_ and N_3_ are number of sperm bound on Location no. 1, 2, and 3, respectively. The binding index was calculated separately for each color.

### *In vivo* trial: microbiology of stored semen samples and fertility post-AI

A field insemination trial was performed at a pig farm located in Rancagua, Chile, to assess the fertilizing capacity of antibiotic-free semen stored at 5 °C compared to semen doses routinely stored at 17 °C with the AB gentamicin sulphate (0.25 g/L). Thirtysix semen pools from 23 mature boars (PIC 337) each consisting of normospermic ejaculates from three boars were split sample-diluted with APrem extender with and without AB. Sperm concentration and volume in semen doses were prepared according the routine use in the breeding organization. After dilution, semen doses were transported within 20 min to the sow farm. Aliquots of diluted semen samples were kept at the laboratory and subjected to the same cooling and storage conditions as samples on the farm. Samples with AB were stored at 17 °C and samples without AB were kept for 6 h at 22–23 °C and then stored at 5 °C in the dark. Directly after dilution (0 h) and at 24 h and 72 h of storage, bacteria counts (colony forming units/mL) were performed after aerobic culture in blood agar (Labser Laboratory, Rancagua, Chile). Estrus was monitored twice daily in presence of a teaser boar. Sows were inseminated two to three times per estrus with the first insemination performed 15–20 h after first detection of estrus. Weaned multiparous sows (parity 1 to 6, n = 666) were post-cervically inseminated using semen doses with 2.2 × 10^9^ spermatozoa in 50 mL stored for 24 to 72 h. Gilts (parity 0) were transcervically inseminated using semen doses with 4 × 10^9^ spermatozoa in 90 mL. Animals were randomly assigned to the two insemination groups according to their entrance in estrus after weaning. Insemination data of 829 sows and gilts (PIC Camborough) were collected over a period of two months.

### Statistical analysis

For data analysis, the Statistical Analysis System for Windows SAS, version 9.4 (SAS Institute Inc., Cary, North Carolina, USA) was used. Depending on the experiment, different (mixed) models were fit to the data: Sperm quality data were analysed with a two-way repeated measures ANOVAs with fixed and random effects. By considering ‘temperature-extender’ as one between-subjects factor with four levels, repeated measures were only present in two dimensions (temperature-extender treatment and time). An interaction term of treatment x time was included in the model to test whether the treatment effect varied according to time points. Various covariance structures were tested for the residuals. According to the Akaike’s and Bayesian’s information criteria a constant correlation between time points (CS) was selected for the former model of motility and membrane integrity. A general unstructured (UN) covariance model was assumed for nonspecific correlation among measurements in the second model of live, low-calcium sperm. A by subject random intercept was added to the models to account for the correlation between observations from the same individuals. If significant effects were observed, post hoc comparison tests regarding the interesting combinations of fixed factors were performed with Bonferroni correction for multiple comparisons. By means of a one-way ANOVA for correlated samples, repeated measurements in explants and in temperature were considered for the analysis the sperm-oviduct binding experiment using a CS covariance structure. Since the within-subjects factor ‘temperature’ contained only two levels, no adjustment for multiplicity was needed. Categorical variables were compared using Chi-square test for comparisons between treatment groups. CFU were allocated to the three categories, CFU = 0, 0 < CFU/ml < 1000 and ≥1000 CFU/ml, and evaluated using the chi-square test or Fisher’s exact test, as appropriate. For dichotomous endpoints (i.e. Non-Return rate and farrowing rate), logistic regression was used with temperature and parity as linear predictors. Due to large sample sizes, count data (i.e. number of offspring in total and number of live offspring) could be evaluated with a two-way ANOVA of aforementioned factors. In general, a p-value of less than 0.05 was considered statistically significant. Data are shown as mean ± standard error of the mean (SEM).

## Results

### Temperature dependence of bacterial growth in semen extender

Growth of test bacteria was screened during storage at 5 °C and 17 °C in extender medium to determine whether a semen storage temperature of 5 °C would limit bacteria growth. Culture medium was used as control medium. After 72°h at 5 °C, bacterial counts remained constant or declined 2- to 20-fold. In contrast, the bacterial population increased at 17 °C storage up to x10^8^ in culture medium and x10^5^ in BTS, respectively (Supplementary SFig. 1).

### *In vitro* trials: Sperm quality in stored semen samples

**Figure 1:**
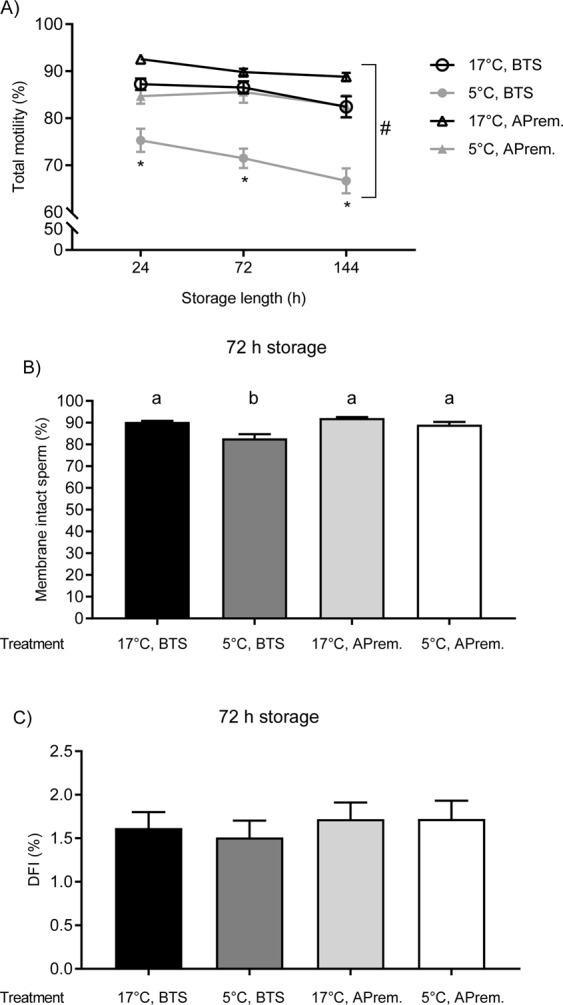
Sperm quality of split semen samples (n = 12 boars) assigned to four groups: (1) BTS extender with antibiotics, storage at 17 °C (17 °C, BTS), (2) BTS extender with antibiotics (AB), storage at 5 °C (5 °C, BTS), (3) AndroStar Premium with AB, storage at 17 C° (17 °C, APrem), (4) APrem without AB, storage at 5 C° (5 °C, APrem). (**A**) Total motile spermatozoa (%; means ± SEM) after 24, 72, and 144 h of storage. (**B**) Plasma membrane and acrosome intact (PI-negative and FITC-PNA negative) spermatozoa (%; means ± SEM) after 72 h of storage. (**C**) DNA fragmentation index (DFI %; means ± SEM) after 72 h of storage. *Values of 5 °C, BTS differ from all other semen groups at a given storage time (p < 0.05).

All effects on sperm motility were statistically significant (temperature-extender treatment: p < 0.001, time: p < 0.001) as well as the interaction effect (p = 0.033), indicating that the treatment has a different effect in storage time points (Supplementary STable 1). Sperm motility did not significantly decline during storage in samples stored in APrem at 17 °C or at 5 °C (Fig. 1A). At all storage time points, sperm motility in 5 °C, APrem was higher than 80% on average (Supplementary STable 2). A statistically significant difference could not be shown between 5 °C/APrem and BTS, 17 °C (p > 0.05). At 144 h, samples in 17 °C, APrem displayed the highest values for sperm motility (88.8 ± 0.9%) and samples in 5 °C/BTS showed the lowest values for this trait throughout storage. The percentage of motile spermatozoa in 5 °C, BTS significantly declined to below threshold values after 144 h of storage compared to 24 h (66.7 ± 2.7 vs. 75.3 ± 2.5; p < 0.001). In the analysis of membrane integrity, temperature-extender treatment showed a significant main effect (p < 0.001) whereas storage time did not show a significant difference (p = 0.238, see Supplementary STable 1). No significant treatment x time interaction was observed (p = 0.272). At 72 h of storage, the proportion of membrane intact spermatozoa did not exhibit a significant difference between 5 °C, APrem (89.2 ± 14.2%) and semen samples stored at 17 °C, BTS (90.3 ± 0.5%) or 17 °C, APrem (92.1 ± 0.5%; Fig. 1B). The percentage of membrane intact spermatozoa in 5 °C, BTS samples (82.8 ± 1.9) was significantly different compared to the other three groups (p < 0.001 for all three comparisons).

The DNA fragmentation index was less than 3% on average in all treatments at 72 h. The effect of treatment was significant (p = 0.048), but did not show significant differences between groups in the post-hoc analysis (Fig. 1C).

**Figure 2:**
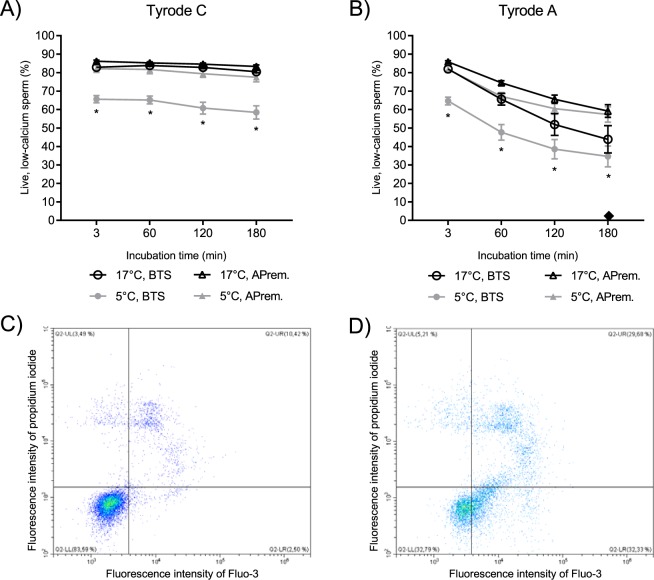
(**A,B**) *In vitro* response of boar semen samples to capacitating conditions during incubation at 38 °C under 5% CO_2_ in Tyrode A medium with bicarbonate (**B**) and to control conditions during incubation at 38 °C in Tyrode C medium without bicarbonate (**A**) as measured by changes in the population of live, low calcium (Propidium iodide negative, Fluo-3 negative) spermatozoa. Semen samples stored for 72 h (n = 6 boars) were assigned to four groups: (1) BTS extender with antibiotics (AB), storage at 17 °C (17 °C, BTS), (2) BTS extender with AB, storage at 5 °C (5 °C, BTS), (3) AndroStar Premium with AB, storage at 17 C° (17 °C, APrem), (4) APrem without AB, storage at 5 C° (5 °C, APrem). ♦: Value after the addition of Ca^2+^-ionophore A23187 in all sample groups. *Values of 5 °C, BTS differ from all other semen groups at a given storage time (p < 0.05). Values declined (p < 0.01) in all semen groups after 60 min of incubation in Tyrode A. (**C,D**) Example of flow cytometry analyses illustrating the shift towards a sperm population with higher fluorescence of the calcium probe Fluo 3 and a concomitant increase of dead spermatozoa (propidium iodide positive) after 60 min incubation in Tyrode A (**D**); this shift is not observed in Tyrode C (**C**). Non-sperm particles were excluded.

Kinetic profiles of calcium influx during incubation in capacitating and non-capacitating media were evaluated to assess the ability of the spermatozoa to respond to capacitation signals. The decrease of the proportion of live low-calcium sperm population (PI-neg/Ca^2+^-neg) is indicative of ongoing destabilization of sperm membranes and is thus a sensitive method to assess storage-induced sublethal effects on sperm function^27,30^. In non-capacitating control medium Tyrode C, both treatment and storage time were statistically significant (temperature-extender treatment: p < 0.001, storage time: p < 0.001, Supplementary STable 3). The interaction effect was non-significant (p = 0.081). The proportion of live low-calcium spermatozoa was significantly lower at all incubation time points in 5 °C, BTS compared to all other treatment groups (p < 0.001 for all comparisons; Fig. 2A). There was a significant main effect for treatment and storage time in capacitating Tyrode A medium (temperature-extender treatment: p < 0.001, storage time: p < 0.001) and no significant interaction effect (p = 0.118, Supplementary STable 3). The proportion of live low-calcium spermatozoa decreased in all sample groups after 60 min compared to 3 min. The 5 °C, BTS samples yielded the lowest values on average compared to all other sample groups at all time points (Fig. 2B). There was no significant difference between 17 °C, BTS, 17 °C, APrem and 5 °C, APrem at any time of incubation (p > 0.05, Supplementary STable 4). As positive control, calcium-influx was inducible after the addition of Ca^2+^ ionophore A23187 in all samples.

### *Ex vivo* trial: Sperm-oviduct binding

**Figure 3:**
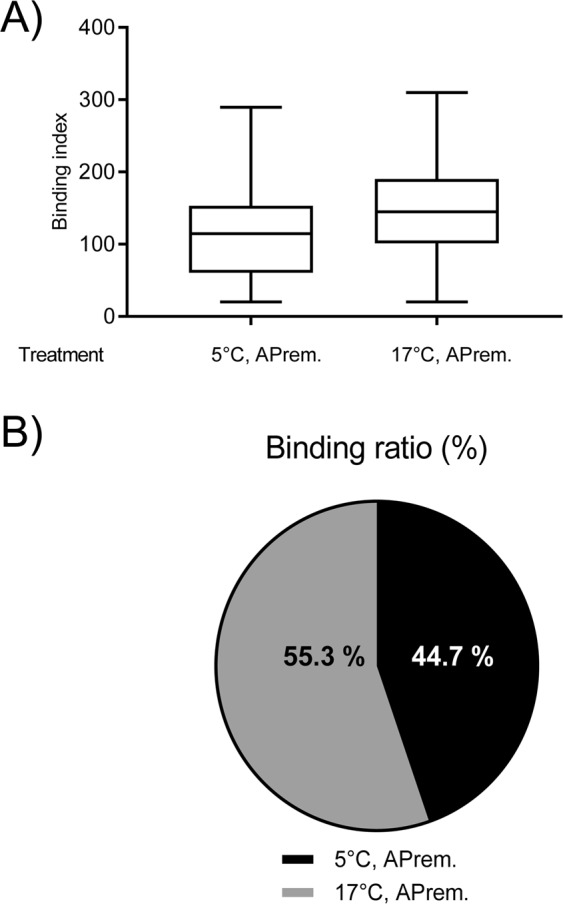
(**A**) Box-whisker plots showing the outcome of competitive sperm binding to oviductal explants of semen samples stored for 72 h either at 5 °C without antibiotics or 17 °C with antibiotics in Androstar Premium (APrem). The number of bound spermatozoa per mm^2^ oviductal surface (binding index) in the two semen groups is illustrated as Box-whisker plots. Values did not indicate statistical significance (p = 0.07). (**A**) The numeric proportion (binding ratio) in the competitive oviduct explant assay is presented as circle-chart (**B**). Values did not indicate statistical significance (p = 0.07). Differential staining was done in spermatozoa taken from the same semen sample and stored either at 5 °C or at 17 °C.

The binding indices resulting from competitive sperm binding to oviductal explant did not significantly differ between 5 °C and 17 °C storage temperature in semen samples extended in APrem (p = 0.072; Fig. 3A). There was a tendency for a skewed sperm binding ratio in favor of samples stored at 17 °C (55.3% bound sperm in samples stored at 17 °C samples vs. 44.7% in samples stored at 5 °C; Fig. 3B).

### *In vivo* trial: Microbiology of stored semen samples and fertility post-AI

#### Microbiology results

**Figure 4:**
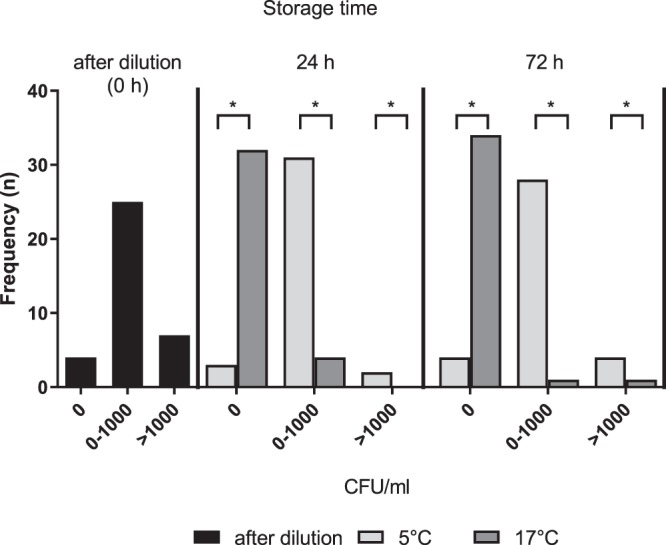
Frequency distribution of semen pools (n = 36) in three classed of bacterial counts (colony forming units (CFU)/ml) initially after dilution in antibiotic (AB)-free Androstar Premium extender (0 h), and after 24 h and 72 h of storage either at 5 °C without AB () or at 17 °C with AB (). Maximum values: 5 °C without AB: 7.6 × 10^3^ (24 h); 17 °C with AB: 1 × 10^4^ (72 h).*Values differ (P < 0.01).

Bacterial counts of semen samples extended in APrem and used for the *in vivo* trial are presented in Fig. 4. Immediately after dilution, 80.6% of the semen pools (0 h) had <10^3^ CFU/ml. After 24 h and 72 h semen storage, the majority of samples contained <10^3^ CFU/ml regardless of presence or absence of AB (with AB/17 °C: 100% and 97.2%; without AB/5 °C: 94.4% and 88.9%, respectively). Maximum bacterial counts in samples with AB/17 °C was 1 × 10^4^ (72 h), and in samples without AB/5 °C it was 7.6 × 10^3^ (24 h). The relation between categorized CFU/ml and storage time was not significant in either temperature group (5 °C: p = 0.445; 17 °C: p = 0.198).

#### Fertility results

Twelve inseminated animals, i.e. eight from the group inseminated with AB/17 °C and five animals from the group inseminated without AB/5 °C, were excluded due to extra-genital illness or death. Parity of females was similar in the two insemination groups (Supplementary STable 5). The percentage of sows versus gilts (parity) did not differ by treatment group (p = 0.21). Semen doses from all 36 pools maintained motility values for 72 h above the threshold of 70°% and were used for AI. Fertility results of 817 females are summarized in Table 1. The fertility traits Non-Return rate, farrowing rate and litter size (total born piglets and live piglets) were all at a high level and did not significantly differ between the animals inseminated with extended semen either containing AB and stored at 17 °C or without AB and stored at 5 °C (p > 0.05, Table 1). Farrowing rates and average numbers of total born piglets of gilts inseminated with AB/17 °C (n = 71) were 94.1% and 14.48 ± 3.35, and of gilts inseminated without AB/5 °C (n = 87) they were 93.1% and 15.04 ± 2.53, respectively.

**Table 1:** Fertility data obtained from insemination of 817 females (659 sows, 158 gilts) with pooled semen from 23 boars (3 boars per pool).

Storage temperature	**17 °C**	**5 °C**	
**Antibiotics**	**Yes**	**No**	
**Females (n)** [Gilts, n]	**406** [71]	**411** [87]	**P-value**
Non-return rate (%)	94.1	94.6	0.902^#^
Farrowing rate (%)	93.1	92.0	0.413^#^
Total piglets born	14.06 ± 0.17	14.51 ± 0.15	0.081^†^
Live piglets born	13.20 ± 0.17	13.70 ± 0.15	0.054^†^

Semen was extended as split samples in AndroStar Premium stored either at 17 °C with antibiotics (0.25 g/L gentamicin sulphate) or at 5 °C antibiotic-free. Data for total and live born piglets are presented as mean and SEM. P-values are results for the factor temperature either of a logistic regression with temperature and parity as linear predictors (#) or a two-way ANOVA for temperature and parity (†).

## Discussion

The limitation of antibiotic use in semen extenders and the concomitant guarantee of microbial safety and high fertilizing capacity of the preserved spermatozoa presents a major challenge in porcine assisted reproduction^1^. The present study provides evidence that boar spermatozoa can be hypothermically stored in an antibiotic-free environment without compromising fertility. The experimental designs were based on two challenges associated with the low-temperature storage and the absence of AB: i). prevention of sperm damage and malfunction due to chilling injury and ii). inhibition of bacterial growth leading to sperm damage and/or affecting the female reproductive tract.

Until now, evidence for the successful application of hypothermic storage of boar semen at supra-zero temperature in large-scale field inseminations is lacking. Cooling induced thermotropic phase transitions of sperm membrane lipids lead to changes in membrane structure, disturbed ion transports and eventually to structural and metabolic disruption of cell function^18,28,37^. The extent of chilling injury can be modified by sperm handling during collection, processing, transport and storage^38^. Considering recently identified semen-processing associated effectors on sperm physiology, data from the present study indicate that boar spermatozoa stored hypothermically at 5 °C maintain motility, membrane and DNA integrity, sperm binding to oviductal epithelium, the response to capacitation stimuli, and ultimately *in vivo* fertility. Essential functional sperm attributes were monitored in several *in vitro* and *ex vivo* assays. Potential damage to these attributes would otherwise remain undetected in an *in vivo* fertility trial due to sperm numbers in the AI doses above threshold values, or dominating farm or female influences^39^. From the results obtained here, it has been demonstrated that 5 °C-storage of boar semen without compromising fertility is practical.

Remarkably, even in BTS-stored samples used as negative control, conventional sperm quality traits were maintained above thresholds for semen use in AI indicating the importance of careful semen processing management.  Particularly a slow cooling achieved by holding times between 17 and 26 °C up to 24 h protects spermatozoa against chilling injury presumably by uptake of membrane stabilizing seminal plasma components^40,41^. The role of long-term semen extenders becomes obvious when comparing *in vitro* sperm data of samples in APrem with the standard short-term extender BTS. Recipes of most commercially marketed semen extenders are not published. However, long-term extenders may contain membrane stabilizers and/or capacitation inhibitors which may prolong shelf life *in vitro*^42^. Cell stabilizing ingredients, such as antioxidants, might hinder the responsiveness of surviving cells to capacitation signals in the female tract^38^. Using a previously established sensitive assay for kinetic assessment of capacitation-associated sperm functionality^33^, we demonstrated that spermatozoa stored in APrem maintain their reactivity to the capacitation stimulant bicarbonate. Noteworthy, destabilization as measured by the progressive loss of live spermatozoa with low calcium occurred slower in APrem compared to BTS controls suggesting a longer survival time in the oviduct.

The binding of spermatozoa to oviductal cells forms a reservoir^43^ which is mediated by carbohydrates on the oviductal cell apical membranes and lectin-like molecules on the sperm surface^44,45^. Several studies have shown that oviductal epithelial cells select boar spermatozoa according to their functional membrane-mediated characteristics^36,46,47^. The slightly reduced binding capacity of spermatozoa stored at 5° observed in the present study may be attributed to chilling-induced membrane lipid alterations. It is suggested that the lower binding capacity can be compensated by a higher sperm number in the semen dose^48^. In the present study, the sperm DNA integrity was not affected by the cold storage, thus compensating effect could become effective. In any case, efficiency of semen use remains high compared to frozen semen where at least a two-fold higher sperm number per semen dose and more inseminations per estrus compared to 17 °C liquid semen storage are required to achieve satisfying fertility outcomes. Noteworthy, in the present *in vivo* trial, sperm numbers per semen dose were equal to the routine farm insemination using semen stored at 17 °C in the presence of AB. As the most relevant finding of this study, fertility of semen stored at 5 °C in absence of antibiotics was not compromised compared to split-semen samples which were conventionally stored at 17 °C in the presence of antibiotics.

Efficient bacteriostasis of four bacteria strains commonly detected in boar ejaculates was achieved in the absence of antibiotics at a 5 °C storage temperature. Bacterial counts in AB-free stored semen was below 10^4^ CFU/ml in all semen samples, and in the majority (88.9%) below 10^3^ CFU/ml. Noteworthy, the highest bacterial count was found in a sample stored with AB at 17 °C, reflecting a typical situation of antibiotic resistance. Logically, depending on temperature tolerance for bacterial multiplication, 17 °C storage bears a higher risk for increased bacterial growth in situations of AB-inefficacy compared to 5 °C AB-free storage. Several studies reported no adverse effects of mesophilic aerobic bacteria on boar sperm quality or fertility if bacteria load is between x10^3^ and x10^7^ CFU/ml^4,5,49–51^. Consequently, there is no rationale for complete elimination of bacteria in pig semen doses. Considering the high numeric exposure to bacteria at natural mating when high semen volumes (200–500 ml) containing up to 10^9^ CFU/ml^14,49^ are transmitted into the female tract, a physiological immunogenic role of commensal bacteria in ejaculates on the uterine milieu deserves to be considered. There are a few specific bacteria that are of concern for causing infectious diseases or loss of reproductive performance in sow herds, including brucellosis, chlamydophilosis and leptospirosis^52^. Regular diagnostic serological testing for brucellosis in boar studs is effective for early detection of this pathogen; therefore, the risk of its occurrence in extended semen is low. The prevalence of *chlamydia* and *leptospira* pathogens in the swine population is largely unknown and may differ among global regions. Strict hygiene measures can greatly aid in controlling the introduction of these pathogens into a boar stud^52,53^. Nonetheless, the efficacy of low temperature semen storage on the growth of specific pathogens should be considered in future studies.

In conclusion, the present study provides strong evidence that hypothermic storage of boar semen at 5 °C allows the elimination of antibiotics in extender used to prepare boar semen doses without affecting fertility. This semen processing strategy can be easily implemented in artificial insemination centers for further evaluation of this novel concept under different farm conditions. Overall, antibiotic-free hypothermic semen storage contributes to bioeconomy in pig AI with a special emphasize on the global antimicrobial defense strategy.

## Supplementary information

Supplementary information

## Data Availability

The datasets generated during and/or analysed during the current study are available from the corresponding author on reasonable request.
