# Supplementary material for: Sperm function in vitro and fertility after antibiotic-free, hypothermic storage of liquid preserved boar semen
Source: Sci Rep. 2019 Oct 14;9:14748. doi: 10.1038/s41598-019-51319-1 (PMC6791940; doi:10.1038/s41598-019-51319-1)
Supplement: Supplementary file 1 — Supplementary information [file 41598_2019_51319_MOESM1_ESM.pdf]

Supplementary information

Sperm function in vitro and fertility after antibiotic-free, hypothermic storage of liquid preserved boar semen

Dagmar Waberski[1], Anne-Marie Luther[1], Benita Grünther[1], Helen Jäkel[1], Heiko Henning[1,2], Charlotte Vogel[3], Wolfgang Peralta[4] & Karl Fritz Weitze[1]

1 Unit for Reproductive Medicine of Clinics/Clinic for Pigs and Small Ruminants, University of Veterinary Medicine Hannover, Bünteweg 15, D-30559 Hannover, Germany

2 Department of Equine Sciences, Faculty of Veterinary Medicine, Utrecht University, Yalelaan 112, 3584 CM Utrecht, The Netherlands

3 Institute for Biometry, Epidemiology and Information Processing (IBEI), University of Veterinary Medicine Hannover, Bünteweg 2, D-30559 Hannover, Germany

4 Agricola Super Ltda., Rancagua, Chile

* Corresponding author: dagmar.waberski@tiho-hannover.de

Supplemental Figure S1
Bacteria growth in culture media and antibiotic-free BTS semen extender after storage for 72 h at 5°C and 17°C. Sterile media were inoculated with 10^3 CFU (colony forming units)/ml with four typical mesophilic gram-negative bacteria strains isolated from boar semen.

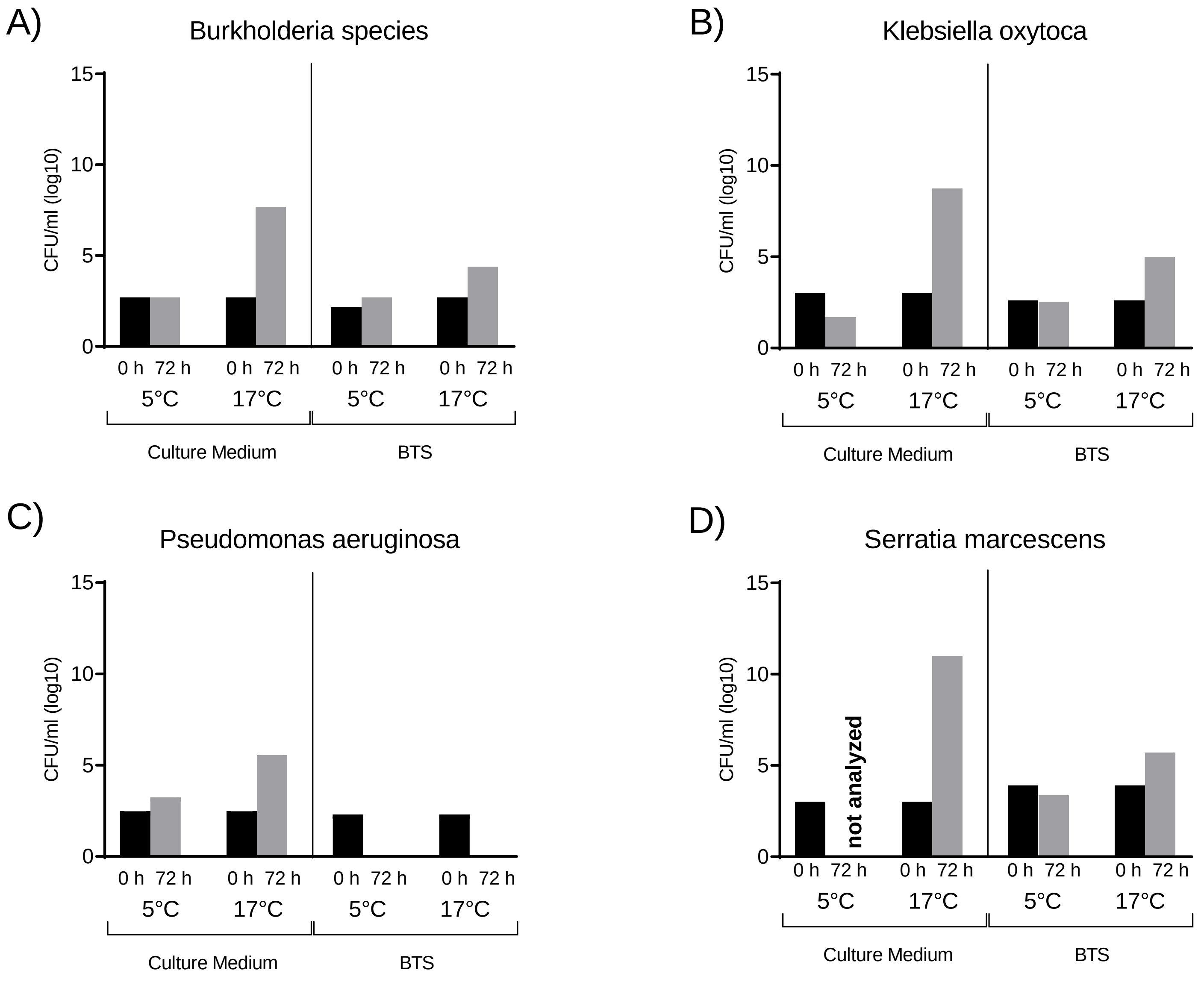

Supplemental Table S1:

Results of the two-way repeated measures ANOVA and selected pairwise comparisons within temperature/extender treatment (A) and storage time point (B). Global p values of the two-way repeated measures ANOVA are reported in brackets (trt = temperature-extender treatment, time = storage time, trt x time = temperature-extender x time interaction). Bonferroni post-hoc tests were carried out within each level of temperature-extender treatment and each level of storage time. Estimated differences are reported with 95% confidence intervals. MI = Membrane integrity (propidium iodide and FITC-PNA negative sperm), DFI= DNA fragmentation index, *P ≤ 0.05, **P ≤ 0.01, ***P ≤ 0.001.

A)

**Comparison**	**24 h**	**72 h**	**144 h**
**Motility %** (trt: p<0.001, time: p<0.001, trt x time: p=0.033)			
17/APrem - 17/BTS	5.3 [-1.2, 11.8]	3.3 [-3.2, 9.8]	6.4 [-0.1, 12.9]
17/APrem - 5/APrem	7.9 [1.4, 14.3]**	4.3 [-2.2, 10.8]	6.2 [-0.3, 12.7]
17/APrem - 5/BTS	17.3 [10.8, 23.8]***	18.4 [11.9, 24.9]***	22.1 [15.6, 28.6]***
17/BTS - 5/APrem	2.5 [-4.0, 9.0]	1.0 [-5.5, 7.5]	-0.2 [-6.7, 6.3]
17/BTS - 5/BTS	12.0 [5.5, 18.5]***	15.1 [8.6, 21.6]***	15.7 [9.2, 22.2]***
5/APrem - 5/BTS	9.4 [2.9, 15.9]***	14.1 [7.6, 20.6]***	15.9 [9.4, 22.4]***
**MI %** (trt: p<0.001, time: p=0.238, trt x time: p=0.0.272)			
17/APrem - 17/BTS	0.7 [-3.8, 5.2]	1.8 [-2.7, 6.2]	2.1 [-2.4, 6.6]
17/APrem - 5/APrem	1.0 [-3.5, 5.5]	2.9 [-1.5, 7.4]	1.9 [-2.6, 6.4]
17/APrem - 5/BTS	4.9 [0.4, 9.4]*	9.3 [4.8, 13.8]***	5.7 [1.2, 10.2]**
17/BTS - 5/APrem	0.3 [-4.2, 4.8]	1.2 [-3.3, 5.7]	-0.2 [-4.6, 4.3]
17/BTS - 5/BTS	4.1 [-0.3, 8.6]	7.6 [3.1, 12.1]***	3.6 [-0.9, 8.1]
5/APrem - 5/BTS	3.9 [-0.6, 8.3]	6.4 [1.9, 10.9]***	3.8 [-0.7, 8.3]
**DFI** (trt: p=0.048)			
17/APrem - 17/BTS	.	0.1 [-0.1, 0.3]	.
17/APrem - 5/APrem	.	-0.0 [-0.2, 0.2]	.
17/APrem - 5/BTS	.	0.2 [-0.0, 0.4]	.
17/BTS - 5/APrem	.	-0.1 [-0.3, 0.1]	.
17/BTS - 5/BTS	.	0.1 [-0.1, 0.3]	.
5/APrem - 5/BTS	.	0.2 [-0.0, 0.4]	.

B)

Comparison	17/APrem	17/BTS	5/APrem	5/BTS
**Motility %**				
24 h – 72 h	2.7 [-1.9, 7.4]	0.7 [-3.9, 5.3]	-0.9 [-5.5, 3.8]	3.8 [-0.8, 8.4]
24 h – 144 h	3.8 [-0.9, 8.4]	4.8 [0.2, 9.5]*	2.1 [-2.5, 6.8]	8.6 [4.0, 13.2]***
72 h – 144 h	1.0 [-3.6, 5.6]	4.1 [-0.5, 8.8]	3.0 [-1.6, 7.6]	4.8 [0.2, 9.4]*
**MI %**				
24 h – 72 h	-1.3 [-5.7, 3.2]	-0.2 [-4.7, 4.2]	0.7 [-3.8, 5.1]	3.2 [-1.3, 7.7]
24 h – 144 h	-1.4 [-5.8, 3.1]	-0.0 [-4.5, 4.4]	-0.5 [-4.9, 4.0]	-0.5 [-5.0, 3.9]
72 h – 144 h	-0.1 [-4.6, 4.4]	0.2 [-4.2, 4.7]	-1.1 [-5.6, 3.3]	-3.7 [-8.2, 0.7]

17/APrem: Semen stored at 17°C in AndroStar Premium extender with antibiotic.
17/BTS: Semen stored at 17°C in BTS extender with antibiotic.
5/APrem: Semen stored at 5°C in AndroStar Premium extender without antibiotic.
5/BTS: Semen stored at 5°C in BTS extender with antibiotic.

Supplemental Table S2:

Sperm quality by temperature/extender treatment and storage time point (24 h, 72 h, 144 h). All results (n=12) are mean ± SEM. DFI was measured at 72 h only. MI = Membrane integrity (propidium iodide and FITC-PNA negative sperm), DFI= DNA fragmentation index.

	**24 h**	**72 h**	**144 h**
**Motility %**			
17/APrem	92.6 ± 0.9	89.8 ± 0.7	88.8 ± 0.9
17/BTS	87.2 ± 1.29	86.5 ± 1.39	82.4 ± 2.29
5/APrem	84.7 ± 1.69	85.6 ± 2.39	82.6 ± 2.39
5/BTS	75.3 ± 2.5	71.5 ± 2.1	66.7 ± 2.79
**MI %**			
17/APrem	90.8 ± 1.2	92.1 ± 0.5	92.2 ± 0.5
17/BTS	90.1 ± 1.0	90.3 ± 0.5	90.1 ± 0.7
5/APrem	89.8 ± 1.2	89.2 ± 1.2	90.3 ± 0.9
5/BTS	86.0 ± 1.5	82.8 ± 1.9	86.5 ± 1.1
**DFI %**			
17/APrem	.	1.7 ± 0.2	.
17/BTS	.	1.6 ± 0.2	.
5/APrem	.	1.7 ± 0.2	.
5/BTS	.	1.5 ± 0.2	.

17/APrem: Semen stored at 17°C in AndroStar Premium extender with antibiotic.
17/BTS: Semen stored at 17°C in BTS extender with antibiotic.
5/APrem: Semen stored at 5°C in AndroStar Premium extender without antibiotic.
5/BTS: Semen stored at 5°C in BTS extender with antibiotic.

Supplemental Table S3:

Results of the two-way repeated measures ANOVA and selected pairwise comparisons of live, low calcium (propidium iodide and Fluo-3-AM negative) spermatozoa (%) within temperature/extender treatment (A) and incubation time (B). Global p values of the two-way repeated measures ANOVA are reported in brackets (trt = temperature-extender treatment, time = storage time, trt x time = temperature-extender x time interaction). Bonferroni post-hoc tests were carried out within each level of temperature-extender treatment and each level of storage time. Estimated differences are reported with 95% confidence intervals. *P ≤ 0.05, **P ≤ 0.01, ***P ≤ 0.001.

A)

Comparison	3 min	60 min	120 min	180 min
**Tyrode C** (trt: p<0.001, time: p<0.001, trt x time: p=0.081)				
17/APrem - 17/BTS	3.2 [-4.0, 10.4]	1.4 [-6.6, 9.3]	1.8 [-7.8, 11.4]	2.8 [-8.4, 14.0]
17/APrem - 5/APrem	4.0 [-3.2, 11.2]	3.6 [-4.4, 11.5]	5.2 [-4.4, 14.8]	5.8 [-5.4, 17.0]
17/APrem - 5/BTS	20.5 [13.3, 27.7]***	20.1 [12.1, 28.0]***	23.8 [14.2, 33.4]***	24.9 [13.7, 36.1]***
17/BTS - 5/APrem	0.8 [-6.4, 8.0]	2.2 [-5.8, 10.1]	3.4 [-6.2, 13.0]	3.0 [-8.2, 14.2]
17/BTS - 5/BTS	17.3 [10.1, 24.5]***	18.7 [10.8, 26.7]***	22.0 [12.4, 31.6]***	22.1 [10.9, 33.3]***
5/APrem - 5/BTS	16.5 [9.3, 23.7]***	16.5 [8.6, 24.5]***	18.7 [9.1, 28.3]***	19.1 [7.9, 30.3]***
**Tyrode A** (trt: p<0.001, time: p<0.001, trt x time: p=0.118)				
17/APrem - 17/BTS	3.8 [-3.1, 10.8]	8.9 [-5.4, 23.2]	13.7 [-8.2, 35.5]	15.3 [-11.3, 41.9]
17/APrem - 5/APrem	4.1 [-2.9, 11.0]	7.4 [-6.9, 21.7]	5.1 [-16.8, 26.9]	1.9 [-24.8, 28.5]
17/APrem - 5/BTS	21.2 [14.2, 28.2]***	26.9 [12.6, 41.1]***	27.1 [5.2, 48.9]**	24.6 [-2.1, 51.2]
17/BTS - 5/APrem	0.2 [-6.8, 7.2]	-1.5 [-15.8, 12.8]	-8.6 [-30.4, 13.2]	-13.4 [-40.1, 13.2]
17/BTS - 5/BTS	17.4 [10.4, 24.3]***	18.0 [3.7, 32.2]**	13.4 [-8.4, 35.2]	9.3 [-17.4, 35.9]
5/APrem - 5/BTS	17.1 [10.2, 24.1]***	19.5 [5.2, 33.7]***	22.0 [0.2, 43.8]*	22.7 [-3.9, 49.3]

B)

Comparison	17/APrem	17/BTS	5/APrem	5/BTS
**Tyrode C**				
3 min – 60 min	0.9 [-1.2, 2.9]	-1.0 [-3.0, 1.1]	0.4 [-1.6, 2.4]	0.4 [-1.6, 2.5]
3 min – 120 min	1.5 [-2.6, 5.6]	0.1 [-4.1, 4.2]	2.6 [-1.5, 6.8]	4.8 [0.7, 9.0]**
3 min – 180 min	2.8 [-2.1, 7.6]	2.4 [-2.5, 7.3]	4.6 [-0.3, 9.4]	7.2 [2.3, 12.0]***
60 min – 120 min	0.6 [-2.7, 4.0]	1.1 [-2.3, 4.4]	2.2 [-1.1, 5.6]	4.4 [1.1, 7.7]**
60 min – 180 min	1.9 [-2.2, 6.0]	3.4 [-0.7, 7.5]	4.2 [0.1, 8.3]*	6.7 [2.6, 10.8]***
120 min – 180 min	1.3 [-1.0, 3.6]	2.3 [0.0, 4.6]*	1.9 [-0.3, 4.2]	2.3 [0.1, 4.6]*
**Tyrode A**				
3 min – 60 min	11.3 [2.7, 19.9]**	16.4 [7.8, 25.0]***	14.7 [6.1, 23.3]***	17.0 [8.4, 25.6]***
3 min – 120 min	20.2 [6.4, 34.1]***	30.1 [16.2, 43.9]***	21.3 [7.4, 35.1]***	26.1 [12.3, 39.9]***
3 min – 180 min	26.7 [9.3, 44.0]***	38.1 [20.8, 55.5]***	24.4 [7.1, 41.8]***	30.0 [12.7, 47.4]***
60 min – 120 min	8.9 [1.5, 16.3]**	13.7 [6.3, 21.1]***	6.6 [-0.8, 14.0]	9.1 [1.7, 16.5]**
60 min – 180 min	15.3 [4.2, 26.5]***	21.7 [10.6, 32.8]***	9.8 [-1.4, 20.9]	13.0 [1.9, 24.1]**
120 min – 180 min	6.4 [0.7, 12.1]*	8.0 [2.4, 13.7]***	3.2 [-2.5, 8.8]	3.9 [-1.8, 9.6]

17/APrem: Semen stored at 17°C in AndroStar Premium extender with antibiotic.
17/BTS: Semen stored at 17°C in BTS extender with antibiotic.
5/APrem: Semen stored at 5°C in AndroStar Premium extender without antibiotic.
5/BTS: Semen stored at 5°C in BTS extender with antibiotic.

Supplemental Table S4:

Changes in live, low calcium (propidium iodide and Fluo-3-AM negative) spermatozoa (%) by temperature/extender treatment and storage time point. All results (n=6) are mean ± SEM.

	**3 min**	**60 min**	**120 min**	**180 min**
**Tyrode C**				
17/APrem	86.1 ± 0.6	85.3 ± 0.7	84.6 ± 0.6	83.3 ± 0.9
17/BTS	82.9 ± 0.7	83.9 ± 0.5	82.8 ± 0.6	80.5 ± 0.7
5/APrem	82.1 ± 1.9	81.7 ± 2.3	79.4 ± 2.0	77.5 ± 2.5
5/BTS	65.6 ± 2.0	65.2 ± 2.1	60.8 ± 3.2	58.4 ± 3.6
**Tyrode A**				
17/APrem	85.8 ± 0.7	74.5 ± 1.1	65.6 ± 2.3	59.2 ± 3.5
17/BTS	82.0 ± 0.7	65.6 ± 3.2	51.9 ± 6.0	43.9 ± 7.4
5/APrem	81.8 ± 1.6	67.1 ± 2.1	60.5 ± 3.1	57.3 ± 4.1
5/BTS	64.6 ± 2.1	47.6 ± 4.2	38.5 ± 5.2	34.6 ± 5.7

17/APrem: Semen stored at 17°C in AndroStar Premium extender with antibiotic.
17/BTS: Semen stored at 17°C in BTS extender with antibiotic.
5/APrem: Semen stored at 5°C in AndroStar Premium extender without antibiotic.
5/BTS: Semen stored at 5°C in BTS extender with antibiotic.

Supplemental Table S5:

Distribution of parities of female pigs in the two insemination groups

Insemination group		Parity at the time of insemination							
		**0 (gilts)**	**1**	**2**	**3**	**4**	**5**	**6**	**Total**
**17°C/with AB**									
	**n**	71	63	80	70	46	42	33	405
	**%**	17.5	15.6	19.8	17.3	11.4	10.4	8.2	
**5°C/without AB**									
	**n**	87	78	73	68	41	45	18	410
	**%**	21.2	19.0	17.8	16.6	10.0	11.0	4.4	
**Total**	**n**	158	141	153	138	87	87	51	815*

*Parities of two sows, one in each insemination group, were not recorded and therefore were not considered in the table.

AB: =Antibiotic in semen extender
